# *Gigas‐Cell1* mediated in vivo haploid induction in *Brassica napus*: A step forward for hybrid development and crop improvement

**DOI:** 10.1111/pbi.70215

**Published:** 2025-07-21

**Authors:** Muhammad Zeeshan Mola Bakhsh, Mengyu Lei, Xiaoyu Zhang, Ahmad Ali, Bin Yi

**Affiliations:** ^1^ National Key Laboratory of Crop Genetic Improvement, National Center of Rapeseed Improvement Huazhong Agricultural University Wuhan China; ^2^ Hubei Hongshan Laboratory Wuhan China

**Keywords:** *Gigas Cell1*, haploid induction, hybrid breeding, in vivo

Double haploid technology is widely used to produce homozygous line within 1 year. In Rapeseed (*Brassica napus*) breeding in vitro pollen culture is used to produce haploid plants, which is time consuming and varietal dependent. Recent progress of in vivo haploid induction techniques in various crops through editing different genes, *CENH3, KPL, PLA1, DMP, PLD3* and *POD65* has facilitated successful haploid induction (Yao *et al*., 2018). So far, limited knowledge is available in targeting these gene (*DMP, CENH3*) and haploid induction in rapeseed (Han *et al*., 2024; Li *et al*., 2022). Gigas Cell 1 (*GIG1*, AT3G57860) encodes a protein which allow cell to enter in to second meiotic division. Mutant population reported to produced diploid gametes (d'Erfurth *et al*., 2009). Although *GIG1* has not been reported for any other function, *GIG1* has been identified in many plant species (Figure S1). *GIG1* has 7 copies in rapeseed (Figure S2), among which only two copies (BnaA09, BnaC08) show higher expression in floral buds, and early developing seeds (Figure S3A). We have further studied sub‐cellular localization of *GIG1* by using Nicotiana benthamiana leaf epidermal cells, and we found eGFP signal of *GIG1* in nucleus of cell, which are merged with nucleus marker (Figure S3B–E). To create mutant lines in *B. napus (cv*. Westar), two guide RNAs (gRNA) were designed using CRISPR P.2.0 website, which were present on second exon of BnaA09‐*GIG1* and third exon of BnaC08‐*GIG1* (Figure 1a). Both gRNAs were amplified and cloned in to PKSE401‐eGFP CRISPR‐Cas9 vector (Figure 1c). The plasmid carrying the construct was transferred in to cv. Westar via Agrobacterium tumefaciens (GV3101). We have successfully obtained 24/70 transgenic plants with our vector efficiency of 34.3%, which were further tested for mutation analysis through high throughput mutation detection technique (Hitom). Among the 24 transgenic plants, 9 were mutant at both target sites, while 11 were mutant at single target site 1 or 2 (Table S2), which shows that our vector was 37.5% efficient for double mutation and 45.8% for single mutation, respectively. Among these, only one (*gig1*‐23) was homozygous mutant which produced all aborted seeds, while all other mutants have heterozygous type of mutation (insertions and deletions). There were no phenotypic alterations in *gig1* mutant (Figure S4A,B). However, *GIG1* expression was significantly reduced in *gig1* as compared to WT (Figure 1e). Moreover, Pollen viability among *gig1* and WT was significantly different with more aborted/died pollens in *gig1*. On average, our study observed that WT pollens are 99% viable while *gig1* pollens was about 60% viable (Figure 1g,h) (Table S6), and in vitro pollen growth was also significantly reduced in *gig1* mutant (30% after 24 h) as compared to WT (60% after 16 h) (Figure 1f,i,j) (Table S1).

**Figure 1:**
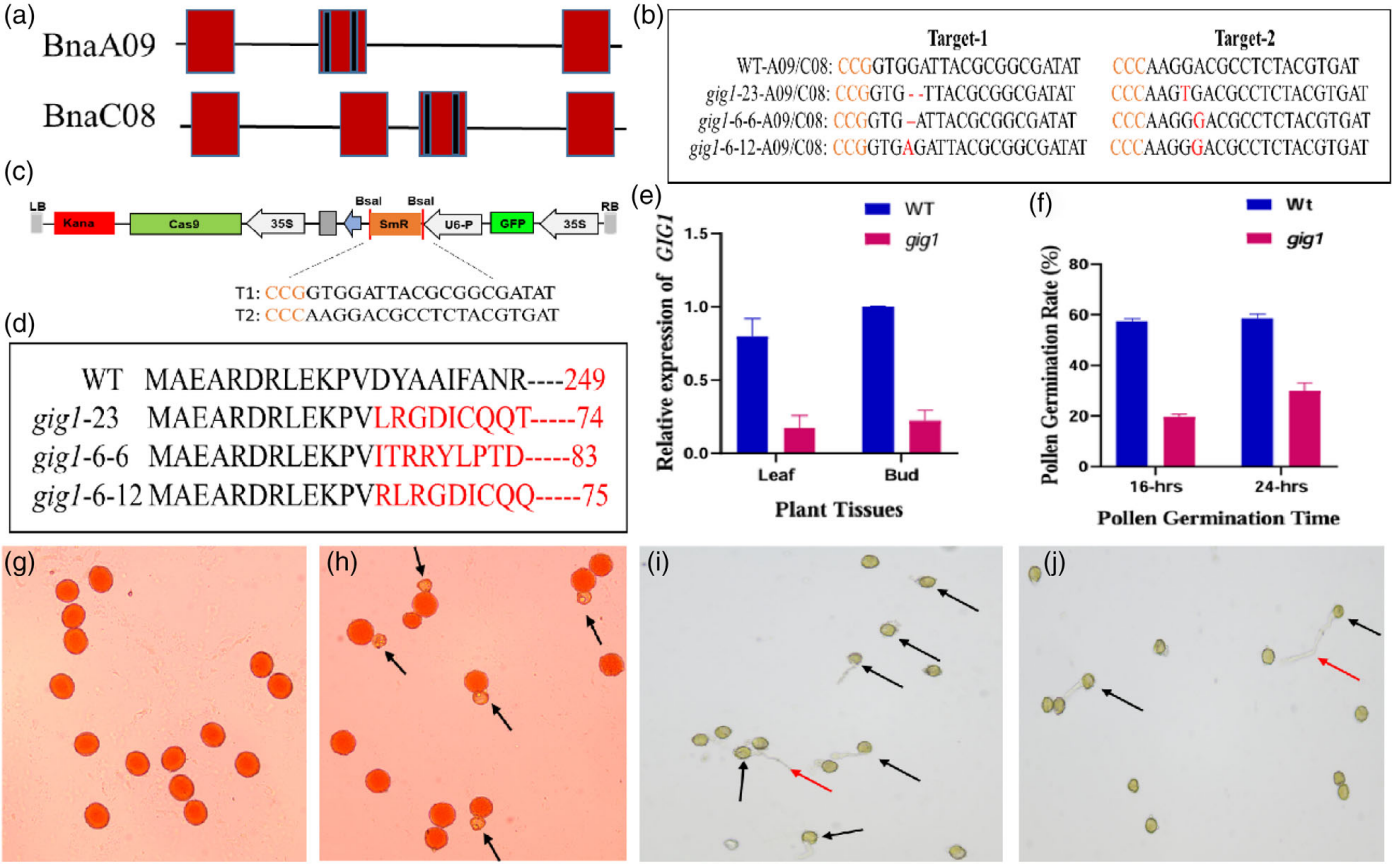
CRISPR Cas9‐mediated *gig1* mutant line as haploid inducer. (a) gRNA location on both targeted copies of *GIG1*. (b) PKSE401‐GFP vector map used in the study. (c) Type of mutation in mutants which were used for haploid induction. (d) Chain of amino acids in different kinds of mutants. (e) Relative expression of *GIG1* in WT and *gig1‐23*. (f) In vitro pollen germination rate. (g, h) Pollen viability of WT and *gig1‐23*, respectively (black arrows indicated aborted pollens). (i, j) In vitro pollen growth of WT and *gig1*‐23, respectively (black arrows indicated germinated pollens, red arrows indicated pollen tube). (k) Haploid induction rate upon crossing with different induced parents.

To test whether T0 and T1 plants of the *gig1* mutant can induce haploidy in *Brassica napus*, *gig1*‐23 (T0), *gig1*‐6‐6 (T1) and *gig1*‐6‐12 (T1) (Figure 1b) were reciprocally crossed with *cv*. Huaye (HY), *cv*. GanA and *cv*. ZS11. F1 seeds from these crosses were grown and tested for the haploid induction rate (HIR) using GFP assay, SSR markers genotyping, flow cytometry, stomata size, chromosome staining and finally whole genome resequencing. Germinated seeds that were negative for GFP were tested for SSR markers. Selected plants from SSR markers were subjected to flow cytometry analysis. Control plants were analysed first and checked their peaks, which were about 7000, already reported by Li *et al*. (2022). Hence, plants that showed a peak of about 3000–3500 were finalized for whole genome resequencing by Ilumina 50K Chip. The plants whose genomes were similar to induced parents were considered haploid plants (Figures 2 and S5, Table S4). The HIR ranged from 1.88% to 2.3%, while no haploid was found in WT and *gig1*‐6‐6 mutant combinations (Table S8). The T1 plant *gig1*‐6‐6 was unable to induce haploidy due to having an 86 amino acids (aa) protein chain of *GIG1*, which is longer than the other two tested mutants *gig1*‐23 and *gig1*‐6‐12 (74aa and 75aa, respectively). Moreover, in all different tested mutant plants, the aa chain changed after 12aa (Figure 1d). Haploid plants were phenotypically and genotypically similar to the induced parent but exhibited more number of stomatas, smaller flower sizes as compared with normal diploid plants, and were male sterile (Figure 2), a common phenomenon of haploid plants. Moreover, our haploid plant produced more buds and flowers as compared to diploid plants, which is a different phenomenon from other reported haploid plants, but the haploid plant buds were smaller as compared to diploid plant buds. The reason for producing more flowers in the haploid is basically a characteristic of the induced *cv*. GanA. Diploid plants exhibited both parents genetic background, which affected the number of flowers in diploid plants, while our haploid plant produced smaller pods as compared to diploid plants upon crossing with WT (Figure S3F). HIR in our study is relatively higher as compared to previously reported rates in *B. napus*. The CENH3‐based HI system HIR ranged between 0.5% and 1.29%, lower than our *gig1* system, while DMP‐based HIR (0.37%–2.5%) (Table S7) in Brassica is almost similar to our *gig1*‐based system, but our system is better due to having ability for reciprocal crosses induction, which in the future plays a significant role in the transfer of sterile cytoplasm. The difference in HIR depends on the type of induced material and seed setting rate, and is directly proportional to the number of aborted seeds (Li *et al*., 2022).

**Figure 2:**
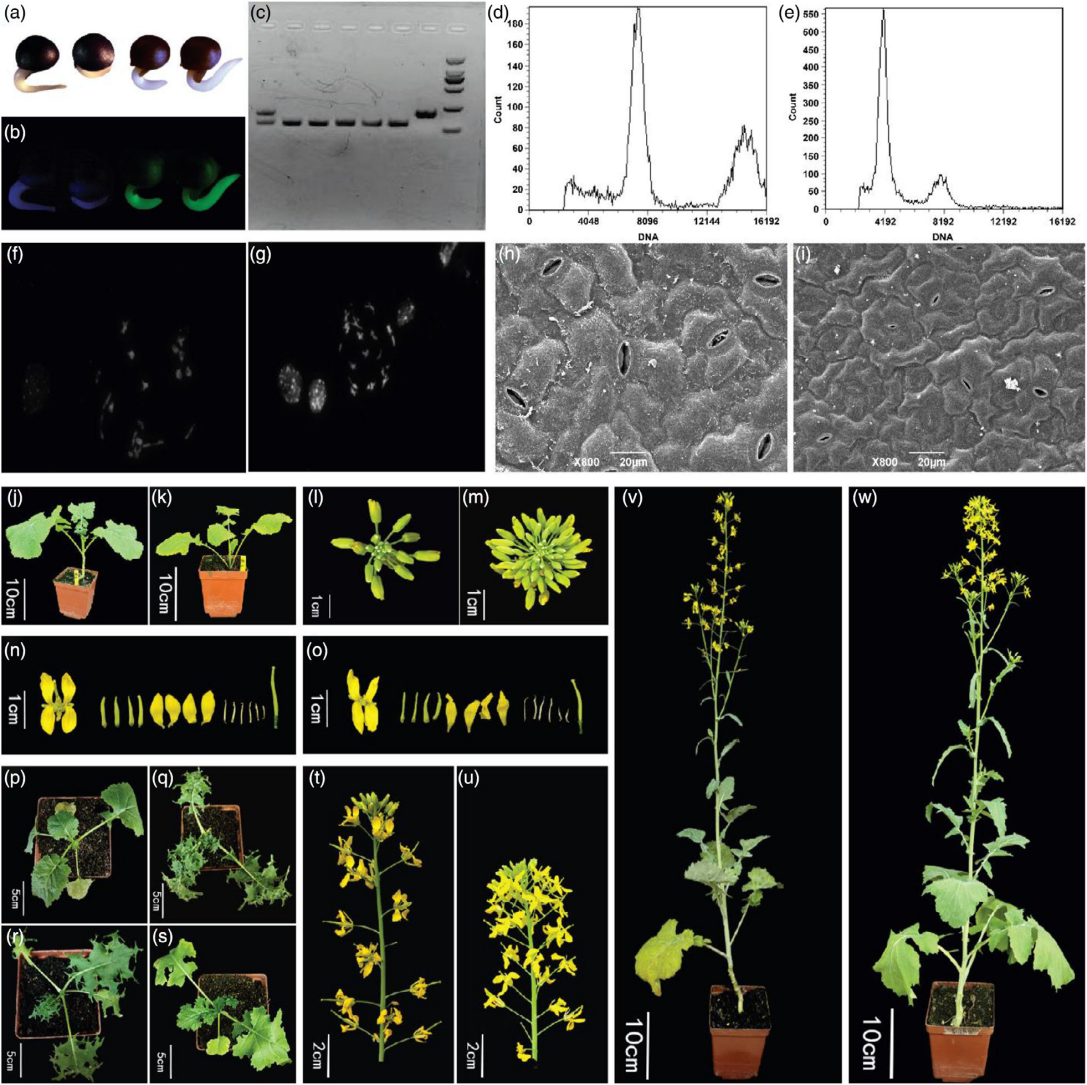
(a–i) (Screening for haploid plants, a‐screening under white light, b‐screening under GFP light, left two seeds without GFP, right two seeds with GFP, seeds without GFP used for further screening, c‐genotyping by using SSR markers, from right side, 2 k marker, inducer parent, induced parent, 4 haploid offspring, 1 diploid offspring, (d, e) flow cytometry analysis of diploid and haploid plant, respectively, (f, g) chromosome staining at meiosis stages of haploid and diploid, respectively, (h, i) number of stomata in diploid and haploid, respectively), (j, k) diploid and haploid young plants, respectively, (l, m) diploid and haploid buds, respectively, (n, o) diploid and haploid flowers with their parts, respectively, (p–s) inducer parent Westar, induced parent Huaye, haploid offspring, diploid offspring, respectively, (t, u) diploid and haploid plant fluorescence, respectively, (v, w) diploid and haploid plants, respectively.

In conclusion, we have successfully established a novel in vivo haploid induction system in *B. napus*, using a new gene *GIG1*. We have created a mutant line which has the ability to induce haploids by using it as a male or female parent. The characteristics of our inducer line as a male or female can be helpful to transfer sterile cytoplasm among different varieties without transferring its own genetic makeup. Furthermore, we added a GFP fluorescent marker to our system, which makes it easier to identify probable haploids at the hypocotyl stage. We predict that during gamete formation, some nullo gametes may be produced due to the mutation of *GIG1*, which causes haploidy. Moreover, our *gig1‐based* HI system is efficient, cheap and without any genotypic barriers compared with traditional DH or in vitro haploid induction systems. In future, we will work on the overexpression of *GIG1*, transferring sterile cytoplasm within one generation and exploring its mechanism of induction using functional genomics or other approaches.

## Author contributions

B.M.Z.M and B.Y. designed the research; B.M.Z.M and M.L. X.Z. and A.A. performed analysis; B.M.Z.M and B.Y. drafted the manuscript. All authors read and approved the final manuscript.

## Supporting information


Appendix S1.

Figure S1–S5.

Table S1–S8.
