# Supplementary material for: Gigas‐Cell1 mediated in vivo haploid induction in Brassica napus: A step forward for hybrid development and crop improvement
Source: Plant Biotechnol J. 2025 Jul 21;23(11):4729–31. doi: 10.1111/pbi.70215 (PMC12576467; doi:10.1111/pbi.70215)
Supplement: Supplementary file 1 — Appendix S1. [file PBI-23-4729-s002.doc]

***Gigas-Cell1* mediated invivo haploid induction in *Brassica napus*: a step forward for hybrid development and crop improvement**

**Material and Methods**

**Plant materials and growth conditions**

The material used for genetic modification was *Brassica napus* (*cv.* westar) while other supporting materials include GanA (Cytoplasmic male sterile), Huaye (HY) and Zhongshuang11 (ZS11) provided by rapeseed engineering center, Huazhong Agricultural University (HZAU) Wuhan, China. All material was grown in field and greenhouse by maintaining 22℃ (16h light/8h dark) of HZAU. Similarly, seedlings of *Nicotiana benthamiana* was grown in greenhouse.

**Identification of homologous genes of *BnaGIG1***

Using gene ID of *Arabidopsis thaliana* (AT3G57860) searched for it homologs in rapeseed by using brassica resource BnIR (yang et al., 2023). We have found seven homologs in westar variety among which only two was expressed (Fig. S2) phylogenetic tree between different crops of *GIG1* was constructed by using geneious software (Fig. S1)

**CRISPR/Cas9-mediated knockout of *BnaGIG1***

A binary vector pKSE401-eGFP with two guide-RNA (gRNA) was constructed (Tang et al., 2018). Two gRNA was designed by using online tool CRISPR P.2.0. Both gRNA was driven by U626 and U629 promoters respectively. While Cas9 was driven by using CaMV 35s promoter. Resulted designed plasmid was transferred in to *Agrobacterium tumefaciens* (GV3101) which was transferred in to 7 days old hypocotyl of *B. napus* (var. westar) by following protocol of Tang et al. (2018). Positive transgenic plants were screened out by using kanamycin antibiotic based growth media and then confirmed by using U626-F, U629-R primer (Table. S5). Selected positive transgenic plants were further tested for type of mutation by using High throughput mutation detection (HI-TOM) technique (Liu et al., 2019). Primers used for HI-TOM are present in supplementary table. S5. All type of knockout lines (Table. S2) were transferred to greenhouse for selfing and crossing.

**Subcellular localization**

Full length coding sequence without stop codon of *GIG1* was amplified by using cDNA of westar, pair of primer used for its amplification was present in table S5. This amplified sequence was cloned in to pMDC83::GFP vector. pMDC83::*GIG1*-GFP and nucleus marker-mCherry were agroinfilterated in to tobacco leaves with help of *Agrobacterium tumefaciens*. The subcellular localization of pMDC83::GFP and nucleus marker mCherry was checked by using LEICA SP8 Germany Confocal microscope. Laser excitation was used at 488nm, 568nm for GFP and mcherry signals respectively.

**Quantitative real-time PCR analysis**

Total RNA was extracted from WT and *gig1* mutant leaves and buds by using RNA extraction kit of shanghai promega catalog no. LS1040 China. All RNA samples were subjected to gDNA remover and cDNA was synthesized by using cDNA synthesis kit by TRANS (catalog no. AE311-02). Quantitative PCR (qPCR) were performed by using ChamQ Blue Universal SYBR qPCR master mix (Q312-02) on Biorad 384wells qPCR. The primers used for qPCR are in supplementary table S5. Three biological replicates were used with Brassica Actin as an internal reference control. Data was analyzed by using 2^-∆∆Ct^ method.

**Pollen Viability and germination**

Fresh mature pollens from WT and T0 mutant were collected from green house with three biological replicates. Pollen was stain by using Acetate Magenta solution and viability was checked under microscope (Nikon, Japan).

Invitro pollen germination was conducted by using pollen growth media (6% polyethylene glycol 6000, 0.01% boric acid, 20% maltose, 0.025% MgSO4•7H2O, 0.001% Vitamin B1, 0.05%Ca(NO3)2•4H2O, 0.03% L-glutathione, 0.03% KCl, 0.001‰ Gibberellin 3) and incubate it in dark at 28°C for 16h and 24h. after specified time pollen germination was observed by using Nikon microscope (Chang et al., 2019). Raw data of pollen germination is present in table S1.

**Screening for Haploid**

F1 progeny from *gig1* mutant and WT crosses was expected to have haploids. All set seeds of different combinations were screened out for haploid progeny. First step of screening, all germinated seeds of F1 were subjected to green fluorescent protein (GFP). All hybrid combinations show GFP signal while without GFP signal was carried out for next step. In second step different SSR markers were used and select only those plants which show band against induced parent. All selected plants in second step were further tested by flow cytometry for ploidy level. Cystain UV ploidy staining solution was used in CyFlow Ploidy analyzer instrument for ploidy analysis (Zhang et al., 2022). These selected plants were further transferred to green house for phenotypic observation. In final step 50K llumina SNP array chip was used for confirmation of genetic background of haploid plants (Xiao et al. 2021). After flowering both haploid and diploid plants bud were subject to chromosome staining and leaves were subjected to stomata size. Compared to diploid, haploid plants were short in height, smaller flower organ, male sterile and similar to induced parent.

**References:**

1. Ahmadli U, Kalidass M, Khaitova LC, Fuchs J, Cuacos M, Demidov D, Zuo S, Pecinkova J, Mascher M, Ingouff M *et al*. 2023. High temperature increases centromere-mediated genome elimination frequency and enhances haploid induction in Arabidopsis. *Plant Communications* **4**: 100507.
2. Chang, H., Zhang, W., Chen, J., Guo, Y., Fang, N., Qiu, Y., Zhou, F., Wu, J., Liu, Z., Liu, S., Wang, Q. (2019). Sugarcane Pollen Germination *in vitro*. Chinese J Trop Crops. 40:2068-2075.
3. Jacquier NMA, Calhau ARM, Fierlej Y, Martinant J-P, Rogowsky PM, Gilles LM, Widiez T. 2023. *In planta* haploid induction by kokopelli mutants. *Plant Physiology* **193**: 182–185.
4. Jiang C, Sun J, Li R, Yan S, Chen W, Guo L, Qin G, Wang P, Luo C, Huang W *et al*. 2022. A reactive oxygen species burst causes haploid induction in maize. *Molecular Plant* **15**: 943–955.
5. Kelliher T, Starr D, Wang W, McCuiston J, Zhong H, Nuccio ML, Martin B. 2016. Maternal haploids are preferentially induced by CENH3-tailswap transgenic complementation in maize. *Frontiers in Plant Science* **7**: 414.
6. Liu, Q., Wang, C., Jiao, X., Zhang, H., Song, L., Li, Y., Gao, C., Wang, K. Hi-TOM: a platform for high-throughput tracking of mutations induced by CRISPR/Cas systems*. (2019).* *Sci China Life Sci*. 62:1-7.
7. Mao Y, Nakel T, Erbasol Serbes I, Joshi S, Tekleyohans DG, Baum T, Groß-Hardt R. 2023. ECS1 and ECS2 suppress polyspermy and the formation of haploid plants by promoting double fertilization. *eLife* **12**: e85832.
8. Ravi M, Kwong PN, Menorca RM, Valencia JT, Ramahi JS, Stewart JL, Tran RK, Sundaresan V, Comai L, Chan SW. 2010. The rapidly evolving centromere-specific histone has stringent functional requirements in *Arabidopsis thaliana*. *Genetics* **186**: 461–471.
9. Tang, T., Yu, X., Yang, H., Gao, Q., Ji, H., Wang, Y., Yan, G., Peng, Y., Luo, H., Liu, K., Li, X., Ma, C., Kang, C., & Dai, C. (2018). Development and Validation of an Effective CRISPR/Cas9 Vector for Efficiently Isolating Positive Transformants and Transgene-Free Mutants in a Wide Range of Plant Species. *Frontiers in plant science*. *9*:1533.
10. Tian S, Zhang J, Zhao H, Zong M, Li M, Gong G, Wang J, Zhang J, Ren Y, Zhang H *et al*. 2023. Production of double haploid watermelon via maternal haploid induction. *Plant Biotechnology Journal* **21**: 1308–1310.
11. Xiao, Q., Wang, H., Song, N., Yu, Z., Imran, K., Xie, W., Qiu, S., Zhou, F., Wen, J., Dai, C., Ma, C., Tu, J., Shen, J., Fu, T., & Yi, B. (2021). The Bnapus50K array: a quick and versatile genotyping tool for Brassica napus genomic breeding and research. *G3 (Bethesda, Md.)*. *11*(10).
12. Yang, Z., Wang, S., Wei, L., Huang, Y., Liu, D., Jia, Y., Luo, C., Lin, Y., Liang, C., Hu, Y., Dai, C., Guo, L., Zhou, Y., & Yang, Q. Y. (2023). BnIR: A multi-omics database with various tools for Brassica napus research and breeding. Molecular plant. 16(4):775–789.
13. Zhang, Y., Huang, X., & Li, W. (2022). Chromosome segregation failure and cytokinesis defect producing unreduced pollen in the diploid rubber tree (*Hevea brasiliensis* (Willd. ex A. Juss.) Müll. Arg.). *J Rubber Res.* **25**:291–298.
14. Zhong Y, Chen B, Wang D, Zhu X, Li M, Zhang J, Chen M, Wang M, Riksen T, Liu J *et al*. 2022a. *In vivo* maternal haploid induction in tomato. *Plant Biotechnology Journal* **20**: 250–252.
15. Zhong Y, Liu C, Qi X, Jiao Y, Wang D, Wang Y, Liu Z, Chen C, Chen B, Tian X *et al*. 2019. Mutation of ZmDMP enhances haploid induction in maize. *Nature Plants* **5**: 575–580.
