# Supplementary material for: Gigas‐Cell1 mediated in vivo haploid induction in Brassica napus: A step forward for hybrid development and crop improvement
Source: Plant Biotechnol J. 2025 Jul 21;23(11):4729–31. doi: 10.1111/pbi.70215 (PMC12576467; doi:10.1111/pbi.70215)
Supplement: Supplementary file 2 — Figure S1–S5. [file PBI-23-4729-s003.docx]

***Gigas-Cell1* mediated invivo haploid induction in *Brassica napus*: a step forward for hybrid development and crop improvement**

**Supplementary Figures**


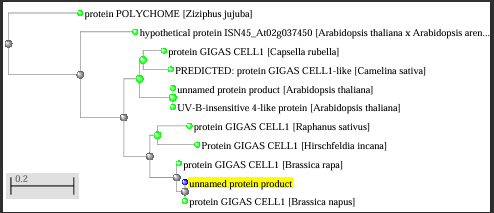


Figure S1. Phylogenetic tree of *GIG1* among different plant species


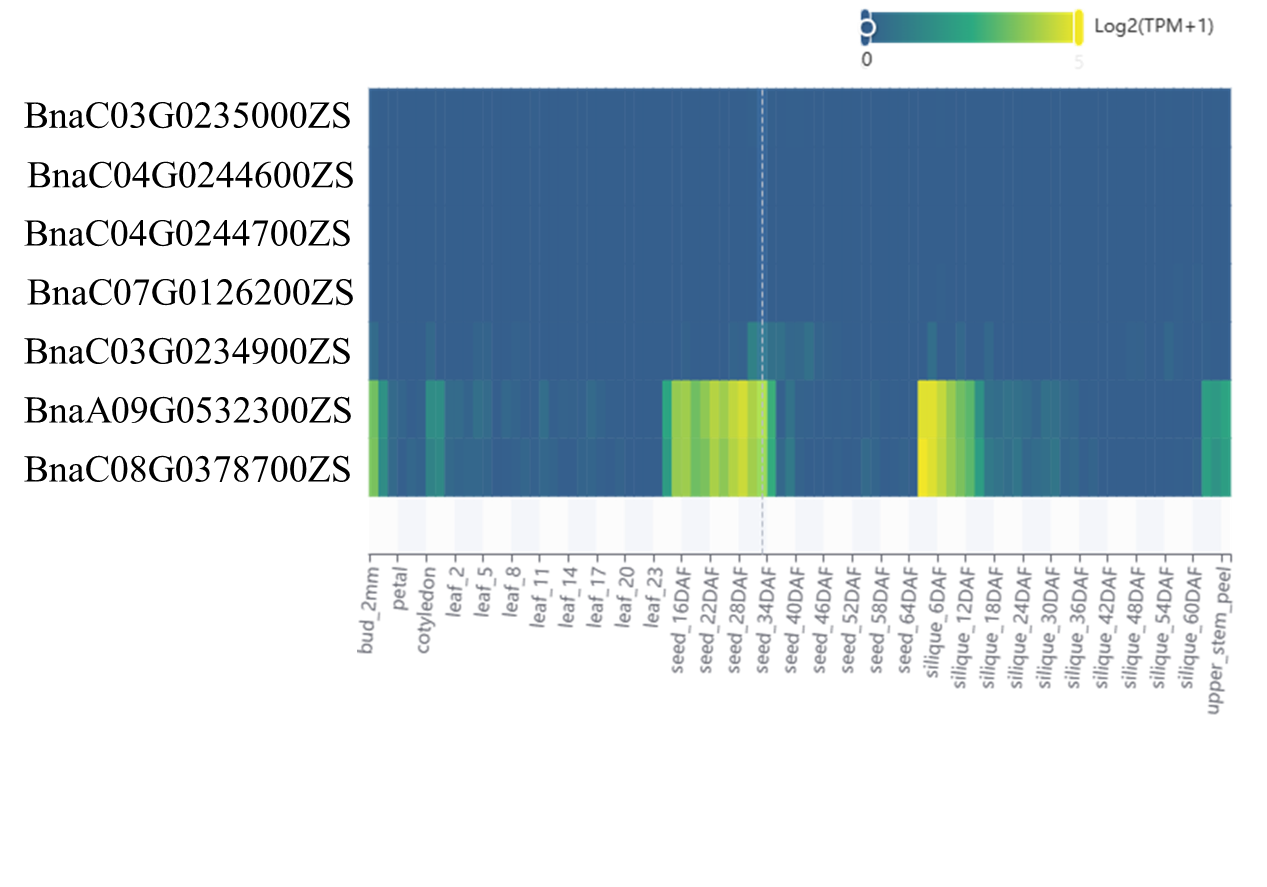


Figure S2. Homologous copies of *GIG1* in *Brassica napus* along with their gene expression in different tissues.

Figure S3. **A-**Relative gene expression of *GIG1* in different plant tissues specifically A09 and C08 homologous chromosome, **B-E**. Subcellular localization of *GIG1* in tobacco leaf cells (B. GIG1-GFP, C. RFP-Marker, D. Bright light, E. Merged), F. Seed setting of diploid plant (left) and haploid plant (right) upon crossing with WT plant.


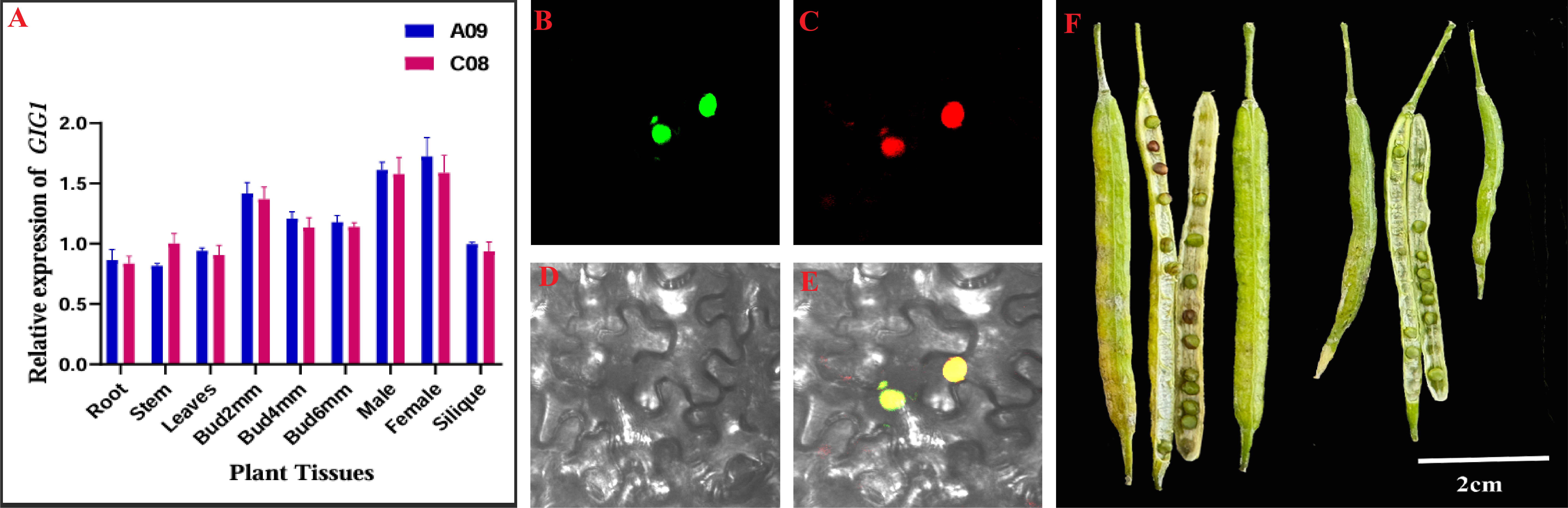

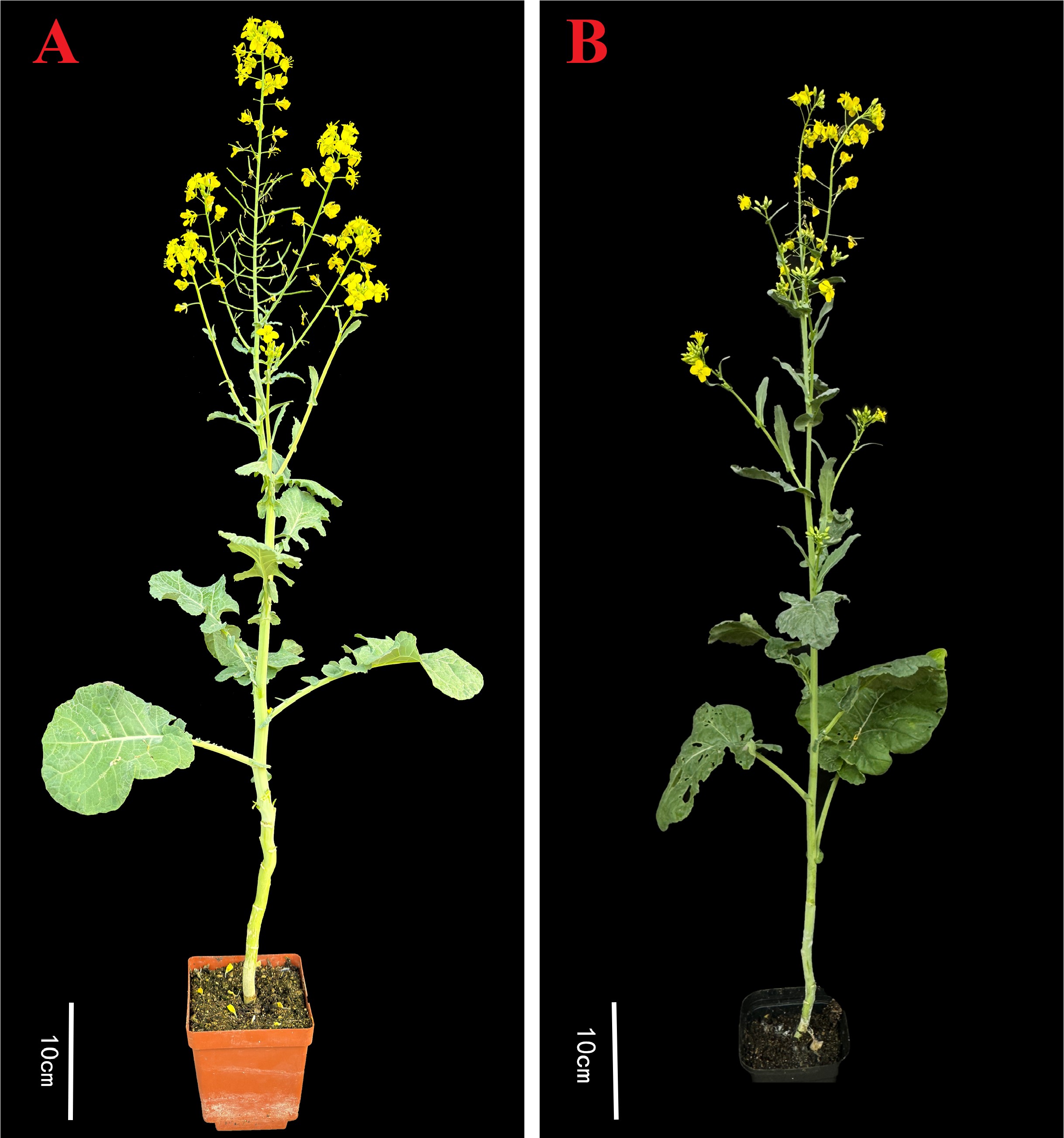


Figure S4. **A-B-**Phenotype of WT and *gig1* mutant (**A.** WT, **B.** *gig1*)

e


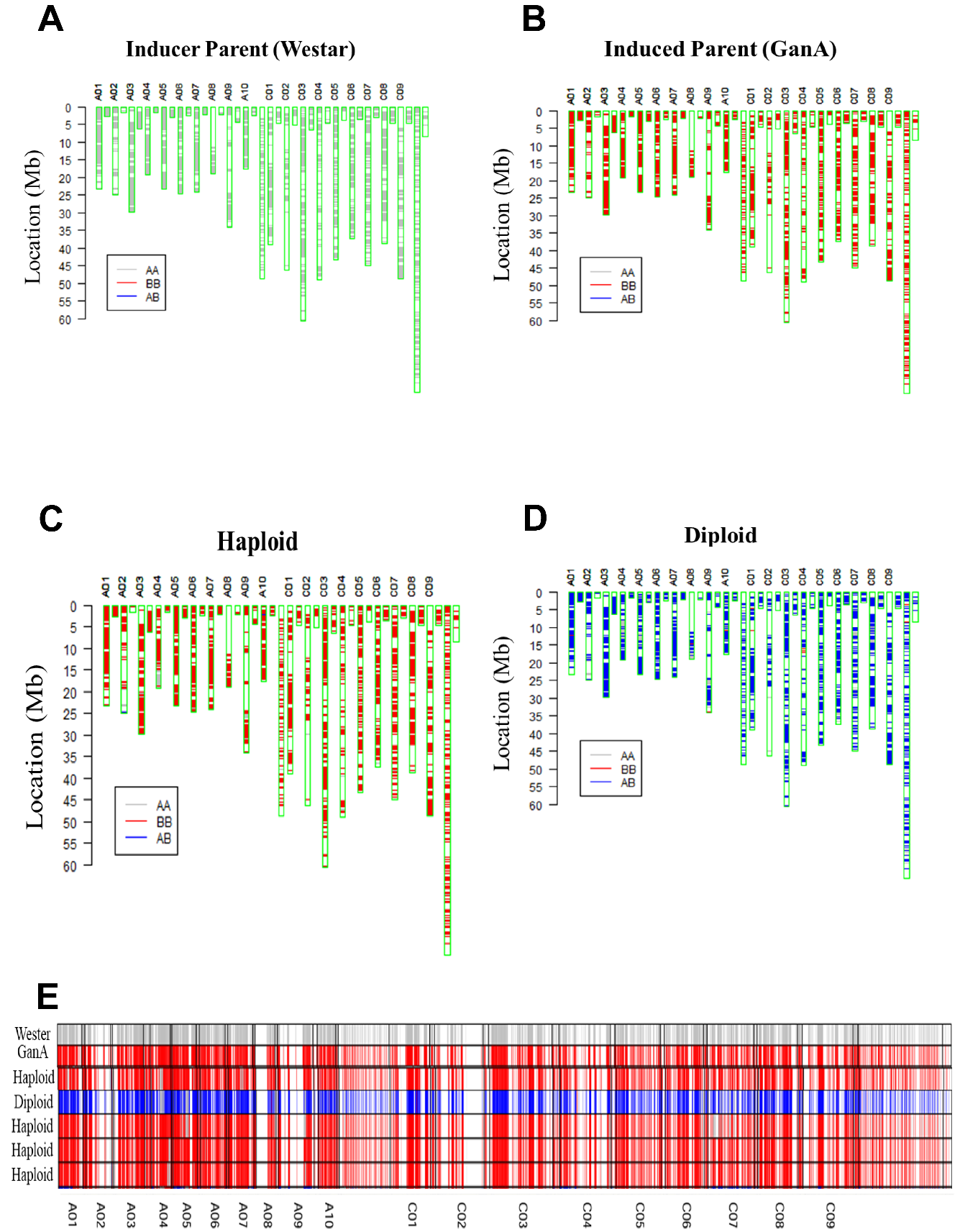


Figure S5. Whole genome resequencing by using 50k-ilumina chip, **A-**Inducer parent (wester), **B-**Induced parent (GanA), **C-**Haploid offspring, **D-**Diploid offspring, **E-** Graphical image of Ilumina 50K chip, Silver color represents Inducer parent, Red color represents induced parents and haploid plants, Blue color represents Diploid plants.
